# Remarkable Reduction of MAP2 in the Brains of Scrapie-Infected Rodents and Human Prion Disease Possibly Correlated with the Increase of Calpain

**DOI:** 10.1371/journal.pone.0030163

**Published:** 2012-01-17

**Authors:** Yan Guo, Han-Shi Gong, Jin Zhang, Wu-Ling Xie, Chan Tian, Cao Chen, Qi Shi, Shao-Bin Wang, Yin Xu, Bao-Yun Zhang, Xiao-Ping Dong

**Affiliations:** State Key Laboratory for Infectious Disease Prevention and Control, National Institute for Viral Disease Control and Prevention, Chinese Center for Disease Control and Prevention, Beijing, People's Republic of China; Ohio State University, United States of America

## Abstract

Microtubule-associated protein 2 (MAP2) belongs to the family of heat stable MAPs, which takes part in neuronal morphogenesis, maintenance of cellular architecture and internal organization, cell division and cellular processes. To obtain insight into the possible alteration and the role of MAP2 in transmissible spongiform encephalopathies (TSEs), the MAP2 levels in the brain tissues of agent 263K-infected hamsters and human prion diseases were evaluated. Western blots and IHC revealed that at the terminal stages of the diseases, MAP2 levels in the brain tissues of scrapie infected hamsters, a patient with genetic Creutzfeldt-Jakob disease (G114V gCJD) and a patient with fatal familial insomnia (FFI) were almost undetectable. The decline of MAP2 was closely related with prolonged incubation time. Exposure of SK-N-SH neuroblastoma cell line to cytotoxic PrP106-126 peptide significantly down-regulated the cellular MAP2 level and remarkably disrupted the microtubule structure, but did not alter the level of tubulin. Moreover, the levels of calpain, which mediated the degradation of a broad of cytoskeletal proteins, were significantly increased in both PrP106-126 treated SK-N-SH cells and brain tissues of 263K prion-infected hamsters. Our data indicate that the decline of MAP2 is a common phenomenon in TSEs, which seems to occur at an early stage of incubation period. Markedly increased calpain level might contribute to the reduction of MAP2.

## Introduction

Prion diseases, also known as transmissible spongiform encephalopathies (TSEs), are a group of fatal neurodegenerative diseases of central nervous system (CNS), including bovine spongiform encephalopathy in cattle, scrapie in sheep and goat, chronic wasting disease (CWD) in deer and elk, and Kuru, Creutzfeldt-Jakob disease(CJD), fatal familial insomnia (FFI) and Gerstmann-Sträussler-Scheinker (GSS) syndrome in humans [1], [2]. They are characterized by progressive neuronal degeneration, neuronal vacuolation and gliosis and the misfolding of normal cellular prion (PrP^C^) into an abnormal form of scrapie prion (PrP^Sc^) that accumulates in CNS [3].

A synthetic peptide corresponding to the amino acid residues 106–126 of prion protein (PrP106–126) is able to induce apoptosis in the primary rat hippocampal cultures [4] and various human neuroblastoma cell lines [5], as well as mouse retinae [6]. Similar to PrP^Sc^, it forms amyloid fibrilar aggregates and to catalyzes the conversion of PrP^C^ to an amyloidogenic form [7]. The similarities between the synthetic PrP106–126 peptide and native PrP^Sc^ to some extents make it a useful mimic in the research of prion and prion diseases.

Microtubules are polarized structures and assemble from heterodimers of α- and β-tubulin in a GTP-dependent fashion. It plays a central role in cellular transport, structural integrity and cellular architecture. The polymerization, stabilization, arrangement of microtubules can be modulated by interactions with a series of microtubule-associated proteins (MAPs)[8]. Microtubule-associated protein 2 (MAP2) belongs to a family of heat stable MAPs, which takes part in neuronal morphogenesis, maintaining cellular architecture and internal organization, cell division and cellular processes [9]. In mammalian brain, MAP2 isoforms have been divided into high-molecular weight MAP2 (HMWMAP2) and low-molecular weight MAP2 (LMWMAP2). HMWMAP2 consists of MAP2a (*Mr.* 280 kDa) and MAP2b (*Mr.* 270 kDa,) that are specially expressed in neurons, while LMWMAP2 includes MAP2c (*Mr.* 70 kDa) and MAP2d (*Mr.* 75 kDa) that are present in glial cells [10]. It has been reported that in the brains of Alzheimer's diseases, the levels of MAP2 are usually decreased [11], [12]. Treatment of Aβ1–42 on C57BL/6J mouse primary cerebral neurons and human neuroblastoma cells induce a reduction of MAP2 [13], [14]. However, the situations and roles of MAP2 in TSE pathogenesis remain unclear.

Many previously studies have repeatedly identified that the levels of tubulin decreased in CNS tissues of naturally occurred or experimental human and animal TSEs [15], [16], [17]. Our previous studies have demonstrated that the CJD-associated PrPs can disrupt the cellular microtubule structure through different pathways [18]. Recently, by screening the transcriptional diversity in the brains of human prion diseases with a commercial mRNA microarray, we found that the expression level of MAP2 is obviously decreased (unpublished data). In this study, we found that MAP2 proteins in the brain tissues of scrapie agent 263K-infected rodents and human genetic prion diseases were almost undetectable at the terminal stages. The decline of MAP2 in the brains of agent 263K-infected hamsters was closely related with prolonged incubation time. These phenomena could be reproduced in a human neuroblastoma cell line SK-N-SH exposed to the synthetic peptide PrP106–126, revealing the decrease of MAP2 and disruption of microtubule structures. Meanwhile, the levels of calpain, which mediates the degradation of a broad of cytoskeletal proteins, were significantly increased in both the brain tissues of scrapie agent 263K-infected hamsters and PrP106–126 treated SK-N-SH cells.

## Materials and Methods

### Ethics statement

Usages of human and animal specimens in this study were approved by the Ethical Committee of National Institute for Viral Disease Prevention and Control, China CDC under protocol 2009ZX10004-101. All signed informed consents have been collected and stored by the China CJD Surveillance Centre. All Chinese golden hamsters were maintained under clean grade. Housing and experimental protocols were in accordance with the Chinese Regulations for the Administration of Affairs Concerning Experimental Animals.

### Samples from scrapie-infected hamsters, human gCJD and FFI patients

Four Chinese golden hamsters inoculated intracerebrally with hamster-adapted scrapie agent 263K and three normal hamsters were enrolled in this study. In addition, the brain samples of the agent 263K-infected hamsters collected on the 20th, 40th, 60th and 80th day post inoculation were included as well. The brains were removed surgically, immediately dissected, then frozen and stored at −80°C until use. The postmortem brains of a G114V gCJD case (47 year-old woman) [19] and a FFI case (26 year-old woman) [20] were also enrolled in this study, which were diagnosed by the experts of Chinese National Surveillance Network for CJD (CNSNC).

### Preparation of brain tissue samples

Brain tissues from experimental hamsters and the regions of thalamus, cingulated gyrus, frontal cortex, parietal cortex, occipital cortex and temporal cortex of human prion diseases were collected and washed with iced TBS (10 mM Tris HCl, 133 mM NaCl, pH 7.4). 10% (w/v) brain homogenates were prepared based on the protocol described previously [21]. Briefly, brain tissues were homogenized in lyses buffer (100 mM NaCl, 10 mM EDTA, 0.5% Nonidet P-40, 0.5% sodium deoxycholate, 10 mM Tris, pH 7.5) containing a mixture of protease inhibitors. The tissue debris was removed with low speed centrifugation at 2000 g for 10 min and the supernatants were collected for further study.

### Western blots

Aliquots of brain homogenates and cell extracts were separated on 6% or 12% SDS-PAGE and electronically transferred to NC membrane. Membranes were blocked with 5% (w/v) non-fat milk powder (NFMP) in 1×Tris-buffered saline containing 0.1% Tween 20 (NFMP-TBST) at room temperature (RT) for 1 h and probed with various primary antibodies at 4°C overnight, including 1∶1000-diluted anti-MAP2 pAb (Cell Signaling), 1∶2000-diluted anti-α-tubulin mAb (Sigma), 1∶1500-diluted anti-Calpain S1 mAb (EPR3324,Abcam), 1∶5000-diluted anti-PrP mAb (3F4, Chemicon) and 1∶2000-diluted anti-β-actin mAb (Santa Cruz), respectively. After washing with TBST, blots were incubated with 1∶5000-diluted horseradish peroxidase (HRP)-conjugated goat anti-mouse IgG or anti-rabbit IgG(Santa Cruz) respectively at RT for 1 h. Blots were developed using Enhanced ChemoLuminescence system (ECL, Amersham Life Sciences, Buckinghamshire, UK) and visualized on autoradiography films.

### Immunohistochemical (IHC) assays

Paraffin sections (5 µm in thickness) were prepared and immunohistochemistry were performed according to the previous protocol. Briefly, sections were quenched for endogenous peroxidases in 3% H_2_O_2_ in methanol for 10 min, pretreated with enzyme digestion antigen retrieval for 1 min. After blocked in 1% normal goat serum, the sections were incubated with 1∶100-diluted mAb for MAP2, 1∶1000-diluted mAb for PrP or 1∶500-diluted mAb for GFAP (Santa Cruz) at 4°C overnight, respectively. Subsequently, the sections were incubated with 1∶250-diluted HRP-conjugated goat anti-mouse secondary antibody (Vector Labs, USA) for 60 min, and visualized by incubation with 3,3′-diaminobenzidine tetrahydrochloride (DAB). Finally, the slices were counterstained with hematoxylin, then mounted in Permount.

### Peptides and chemicals

PrP106-126 (KTNMKHMAGAAAAGAVVGGLG) and scrambled PrP106-126 (SCR) (AVHTGLGAMAALNMVVGGAAGL) were synthesized and purified by Sheng Gong Biological Co (Shanghai, China). The peptides were dissolved in DMSO at a concentration of 50 mM and stored at −80°C.

### Cell treatment and cell viability assays

Human SK-N-SH cells were obtained from Experimental Animal Center of Sun Yat-sen University. Cells were cultured in Dulbecco's modified Eagle's medium (DMEM, Gibco) that contained 10% FBS (Gibco) in a humidified incubator supplied with 5% CO_2_. SK-N-SH cells were seeded on a 96-well plate at a concentration of 10^4^ cells per well and cultured for 24 h. PrP106-126 and SCR were diluted to the desired concentration in DMEM without FBS and added directly to the cells. Cell viability was determined by a commercially Cell Counting Kit (CCK-8, Dojindo, Japan). 10 µl of CCK-8 regent were added to each well and incubated at 37°C for 1 h, until the media turned yellow. Each experiment was performed in triplicate and repeated at least three times with separated cell preparations.

### Immunocytochemical staining

Cells were fixed with formaldehyde (4% paraformaldehyde, freshly depolymerized in 0.1 M sodium phosphate buffer, pH7.4) at RT for 15 min and washed three times in PBS. After blocked with blocking buffer (PBS with 5% FBS and 0.1% Trition X-100) at RT for 1 h, cells were incubated with 1∶50 diluted anti-MAP2 pAb (Cell Signaling) or 1∶200 diluted anti-α-tubulin mAb (Sigma) in dilute solution (PBS with 2% BSA and 0.3% Trition X-100) at 4°C overnight. Cells then were washed and incubated with 1∶200 diluted (v/v) appropriate secondary antibodies (Alexa Fluor anti-mouse 488 or Alexa Fluor anti-rabbit 568, Invitrogen) in dilute solution at RT for another 2 h. After washing, cells were incubated with 0.5 mg/ml DAPI (Invitrogen) at RT for 2 min. Cells were sealed and the images of the targeting proteins were analyzed by microscope (Olympus BX51) and confocal microscopy (Leica ST2, Germany). Density analysis was performed using Image-Pro Plus 5.0, and the mean optical density (MOD) was slope value measured by counting the optical and area values of each positive stained cell in a certain field of view.

### Statistical analyses

Quantitative analysis of immunoblot images was carried out using software Image J. The values of each target blot were evaluated. All data are presented as the mean ± SD. Statistical analysis was performed using the *T* test. Probabilities of less than 0.05 were considered to be statistically significant.

## Results

### Decrease of MAP2 in the brains of 263K prion-infected hamsters at terminal stages

**Figure 1:**
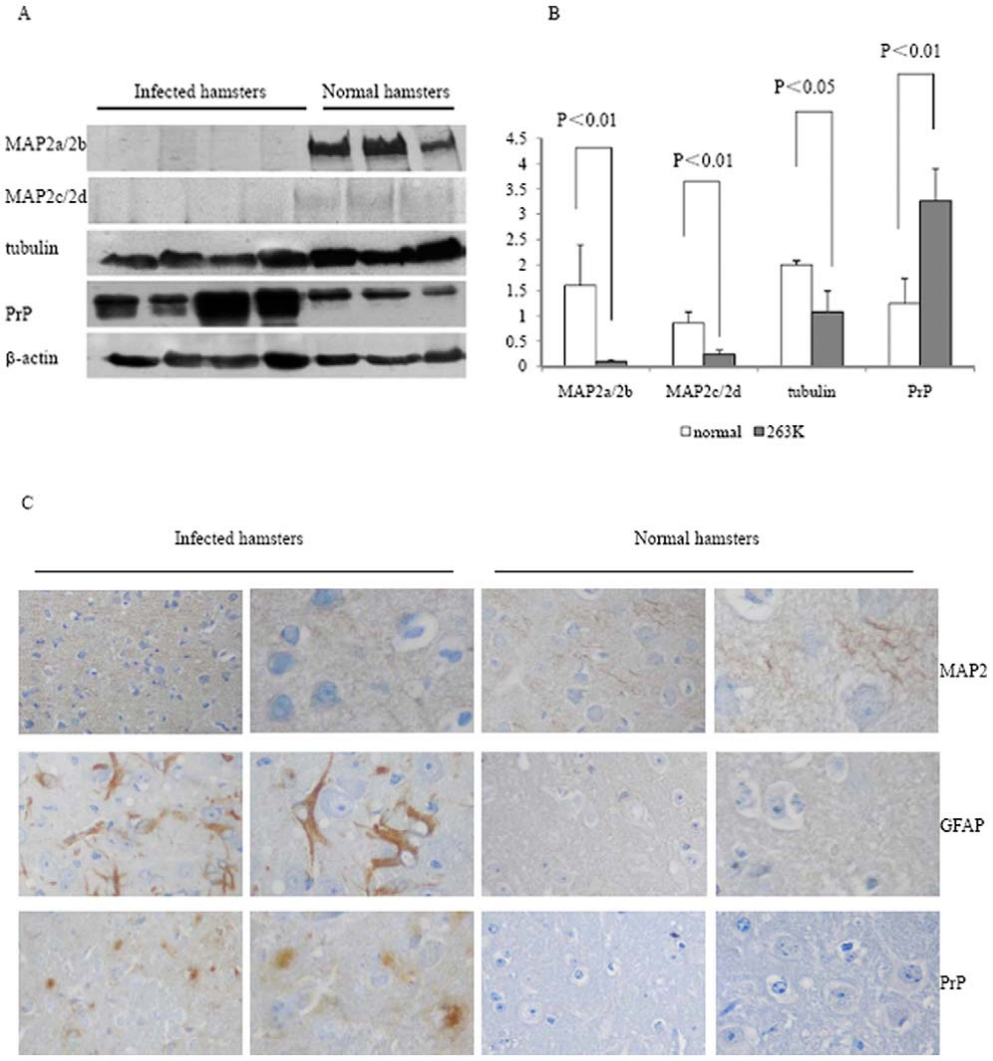
Comparative analyses of the levels of MAP2 in brain tissues of normal and 263K-infected hamsters. A. Western blots. Same amounts of individual brain homogenate were loaded in 6% or 12% SDS-PAGE and various specific immunoblots were marked on the left side of the graphs. B. Quantitative analysis of each gray numerical value of MAP2a/2b, MAP2c, 2d, tubulin and total PrP vs that of individual β-actin. The average values were calculated from four individual infected hamsters or three individual normal hamsters and presented as mean ± SD. Statistical differences compared with controls were illustrated as P<0.05 or P<0.01. C. IHC assays of MAP2, total PrP and GFAP in cortex of normal and 263K-infected hamsters. The magnifications are ×20 in the left row and ×40 in the right row.

To assess the potential changes of MAP2 in the brain tissues of TSE, 10% brain homogenates of four scrapie strain 263K prion-infected hamsters were included in this analysis. The amounts of MAP2, tubulin and total PrP were evaluated by Western blots with specific antibodies. Large amounts of total PrP signals were detected in the brains of 263K prion-infected hamsters, while the signals of MAP2, including MAP2a and 2b with *Mr.* 280 and 270 kDa, and MAP2c and 2d with *Mr.* 70 and 75 kDa, were almost undetectable (Fig. 1A). The amounts of tubulin in the brains of 263K-infected animals also remarkably decreased compared to that of healthy hamsters (Fig. 1A). Relative protein levels were normalized to β-actin control and showed that the mean relative quantities of MAP2a/2b, MAP2c,2d and tubulin in the brains of 263K-infected hamsters were significantly lower, while total PrP protein were significantly higher than those of the healthy ones (P<0.05, Fig. 1B).

To verify the reduction of MAP2 in scrapie-infected animals, the status of MAP2 in the brain tissues of 263K-infected hamsters were analyzed with MAP2-specific immunohistochemistry (IHC). In parallel, PrP^Sc^ deposits and astrogliosis were monitored with PrP-and GFAP-specific IHC. As expected, large quantities of PrP^Sc^ deposits were observed in cortex regions of 263K-infected hamsters but not in normal controls (Fig. 1C, third row from top). More GFAP positively stained long-fibrous like cells were detected in the brain of 263K-infected hamsters, but there were almost no such structures in the normal animals (Fig. 1C, second row). MAP2 positive-stained particles were easily observable in the brains of normal hamsters, but much weaker in that of 263K-infected hamsters (Fig. 1C, first rows). These data indicate that the level of MAP2 in the brains of scrapie experimental rodents is significantly reduced at terminal stages of the disease.

### Alteration of MAP2 isoforms during the incubation time

**Figure 2:**
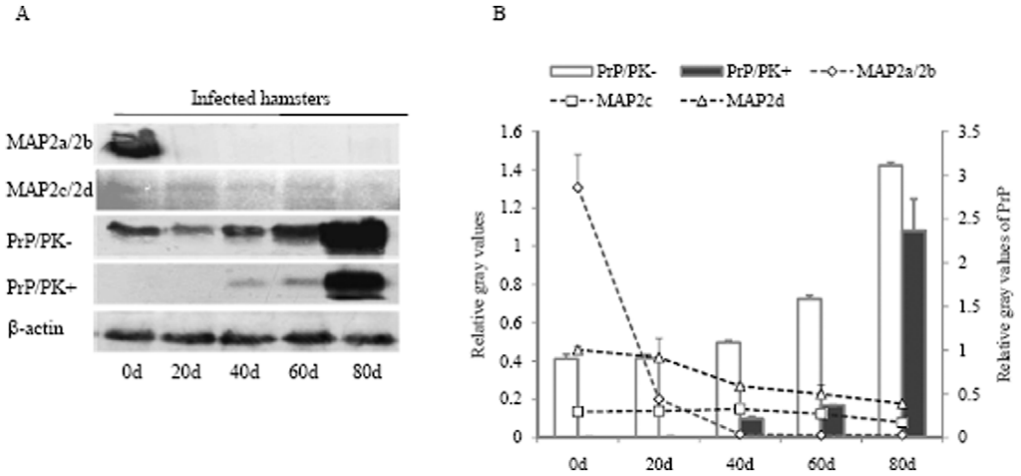
Dynamic analysis of MAP2 and PrP^Sc^ in the brain tissues of normal and 263K-infected hamsters during incubation period. A. Western blots. Same amounts of individual brain homogenate were loaded in 6% or 12% SDS-PAGE. Various specific immunoblots were marked on the left and the time of post-inoculation are showed as days (d) at the bottom. B. Quantitative analysis of each gray numerical value of MAP2a/2b, MAP2c, 2d and PrP^Sc^ vs that of individual β-actin. The average relative gray value is calculated from three independent blots and presented as mean ± S.D.

In order to investigate the dynamic changes of MAP2 isoforms in the brains of scrapie-infected hamsters during incubation period, the expressing level of MAP2 in the brain samples of 263K-infected hamsters collected on the 0th, 20th, 40th, 60th, and 80th days post-inoculation (dpi) was comparatively evaluated by Western blot. Fig. 2A revealed obvious PK-resistant PrP signals (PrP^res^) in the brains of 263K-infected hamsters since 40 dpi, but not in normal controls. The signal intensities of total PrP and PrP^res^ became stronger along with the incubation prolonging, showing a time-dependent manner. In contrast, the signals of MAP2c,2d weakened gradually along with the incubation and that of MAP2a/2b even vanished in the samples of 20 dpi (Fig. 2A). After normalized to respective β-actin, the data revealed a clear time-dependent increase of PrP and PrP^Sc^ and a time-dependent decrease of MAP2 isoforms with prolonging incubation (Fig. 2B).

### Similar reduction of MAP2 was detected in the brains of human prion diseases

**Figure 3:**
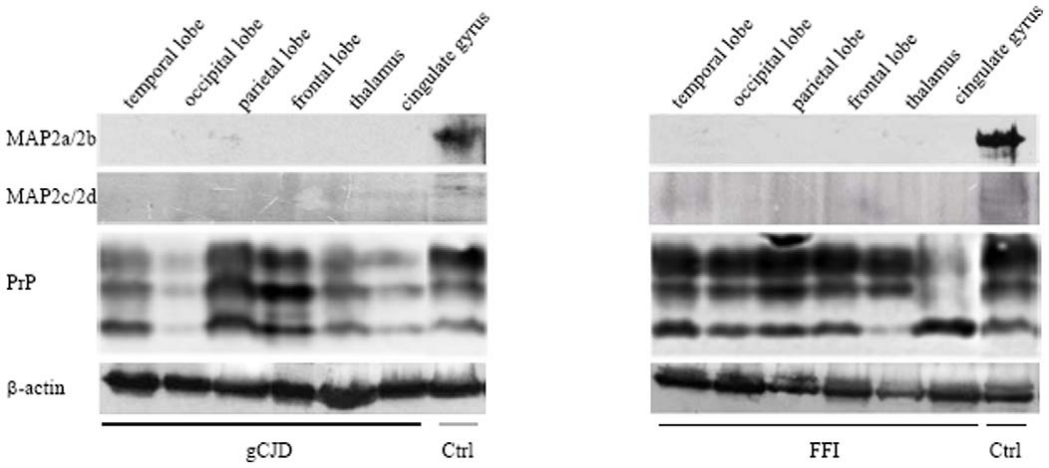
Analyses of the levels of MAP2 in the brain tissues of human prion diseases. A. Western blots of six brain regions of a gCJD patient (left panel) and a FFI patient (right panel). The immunoblots for MAP2, total PrP and β-actin are indicated on the left. Various brain regions are indicated at the top.

Levels of MAP2 expression in postmortem brain from a G114V gCJD patient and a D178N FFI patient were assessed by Western blots, including frontal lobe, parietal lobe, occipital lobe, temporal lobe, thalamus and cingulated gyrus. In line with the observations in 263K-infected hamsters, MAP2 specific signals were barely detectable in all tested brain regions from either G114V gCJD or D178N FFI, but were clearly detectable in controls (Fig. 3). This finding suggests that the reduction of MAP2 in central nervous tissues is a common feature for TSEs.

### PrP106–126 peptide induced the decline of MAP2 in the cultured cells

**Figure 4:**
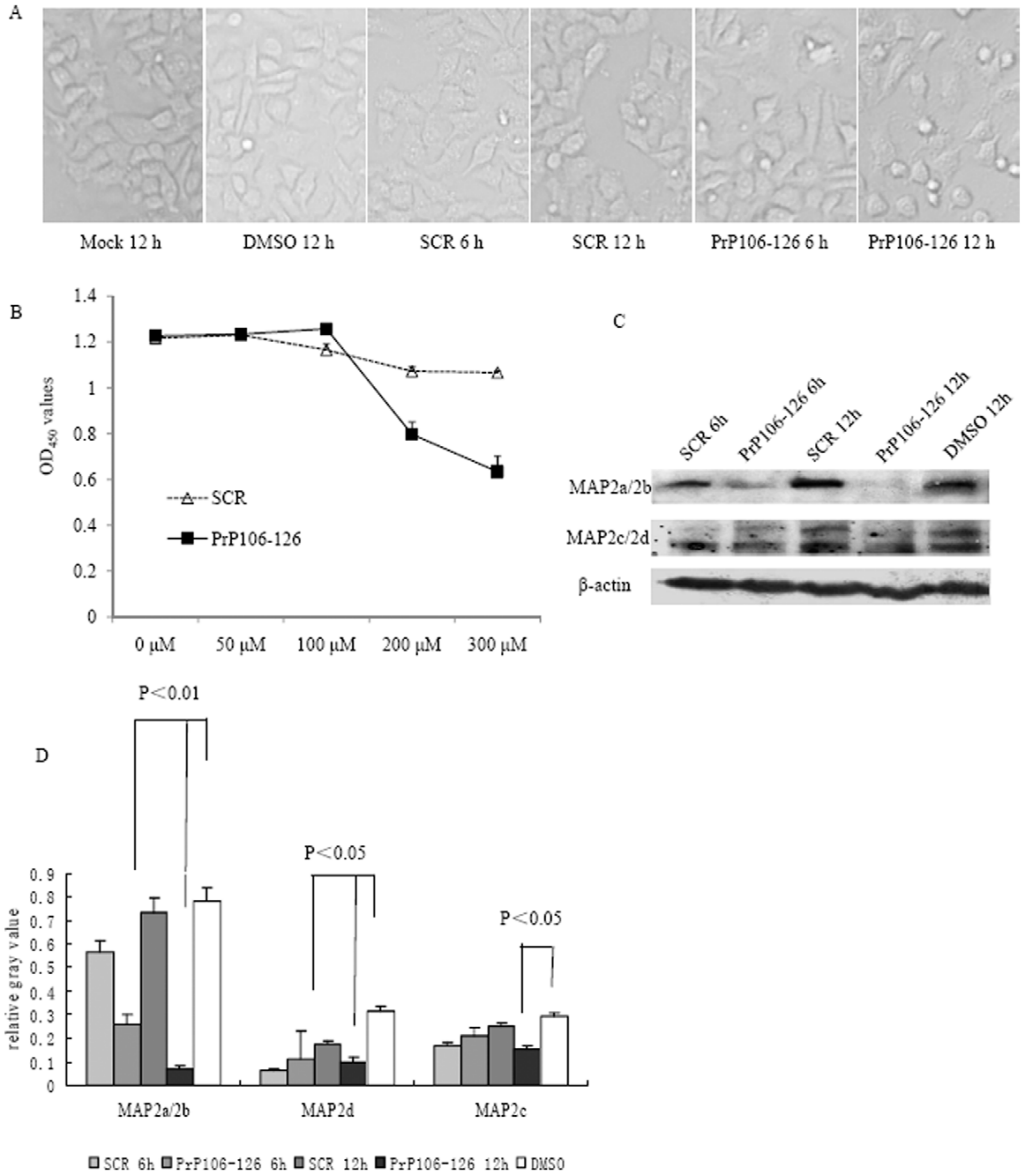
Analyses of the MAP2 levels in SK-N-SH cells exposed to PrP106–126. A. SK-N-SH cells were treated with 200 µM of PrP106–126, scrambled peptide PrP106–126 (SCR) or DMSO for 6 h and 12 h. The treated cells were photographed with a light microscope (×20). B. Cell viability after exposed to different concentrations of PrP106–126 or SCR. The average data of each preparation was calculated based on three independent experiments and represented as mean ± S.D. C. Western blots of MAP2a/2b, MAP2c, 2d and β-actin in SK-N-SH cells after treated with 200 µM of PrP106–126, SCR or DMSO for 6 h and 12 h. D. Quantitative analyses of each gray numerical value of MAP2a/2b, MAP2c, 2d vs that of individual β-actin. The average relative gray value is calculated from three independent blots and presented as mean ± S.D. Statistical differences compared with controls are illustrated as P<0.05 and P<0.01.

The cytotoxic peptide PrP106–126 has been widely used to mimic the pathological features of PrP^Sc^ *in vitro*. To identify the potential influences of PrP106–126 on MAP2 levels in the cultured cells, human neuroblastoma cells SK-N-SH were exposed to different amounts of PrP106–126 or scrambled PrP106–126 (SCR). Morphological assays revealed obvious abnormality on the cells treated with 200 µM PrP106–126 for 12 h (Fig. 4A). Cell viability tests also identified markedly cytotoxicity in the preparations exposed to 200 µM PrP106–126, while the cells exposed to SCR peptide remained almost unchanged (Fig. 4B). Western blots of the cell lysates showed that the levels of MAP2 in cells treated with PrP106–126 for 6 and 12 h were significantly reduced compared to the control groups (DMSO and SCR) (Fig. 4C). Analyse of the relative gray values of each blot after normalization with β-actin revealed a statistically significant decrease of isoforms in PrP106–126 treated cells (Fig. 4D).

**Figure 5:**
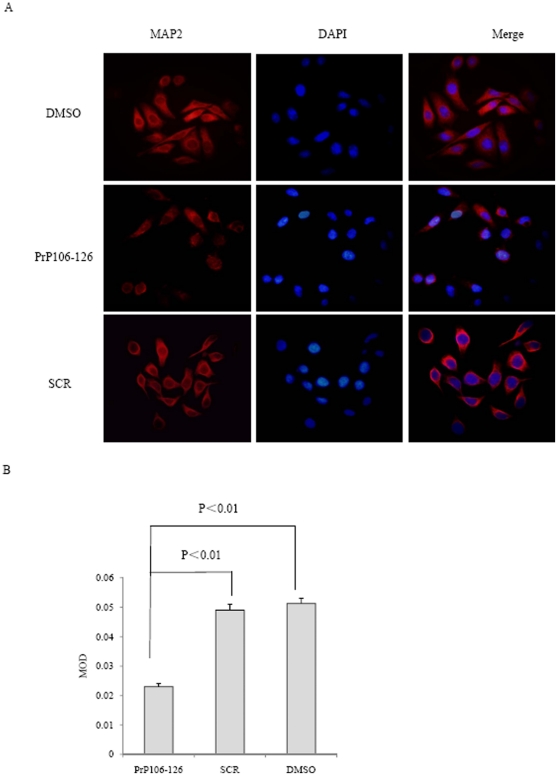
Immunofluorescent assays of MAP2 on PrP106–126 treated SK-N-SH cells. A. Immunofluorescence images of the cells were exposed to DMSO (upper), PrP106–126 (middle) or SCR (lower) for 6 h. The images of MAP2 (red), DAPI (blue) and merge are indicated above. B. Quantitative analysis of fluorescence intensity of MAP2 in the cells. MOD data each preparation is calculated from three independent images and presented as mean ± S.D. Statistical differences is illustrated as P<0.01.

The finding that PrP106–126 reduces cellular MAP2 levels was further verified by immunofluorescence staining. Cells exposed to PrP106–126, SCR peptide and DMSO for 6 h were subjected into immunofluorescent assays with anti-MAP2 antibody. PrP106–126 treatment led to weaker MAP2 stained signals (red color) in the cytoplasm of the cells compared with SCR peptide or DMSO treatment. (Fig. 5A). Comparison of the MOD values among the three preparations showed significant difference (P<0.01) between the groups of PrP106–126 and DMSO, or between PrP106–126 and SCR peptide (Fig. 5B). These data imply that the treatment of PrP106–126 induces an obvious reduction of MAP2 in the cultured cells.

### Treatment of PrP106–126 on SK-N-SH cells did not alter the level of cellular tubulin but destroyed cellular cytoskelet al network

**Figure 6:**
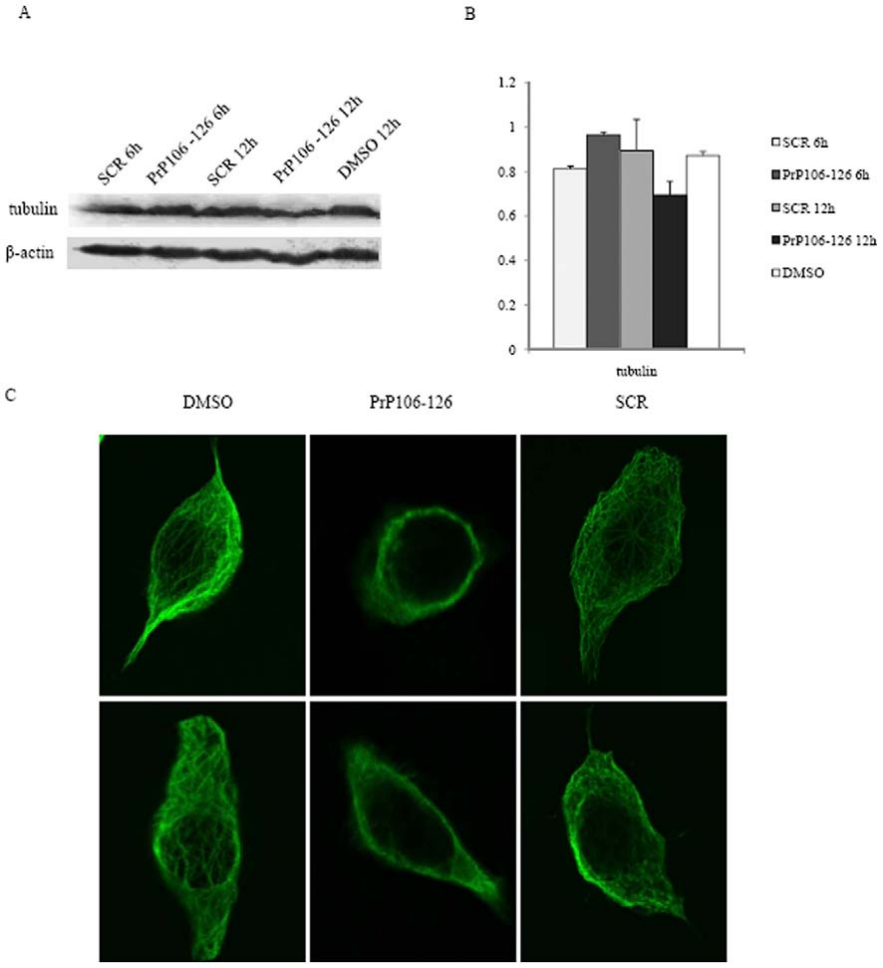
Analyses of the tubulin levels and microtubule structure in cells exposed to DMSO, PrP106–126 or SCR. A. Western blots with anti-α-tubulin mAb. B. Quantitative analyses of each gray numerical value of tubulin vs that of individual β-actin. The average relative gray value is calculated from three independent blots and presented as mean ± S.D. C. Immunofluorescence images of cellular microtubule structure.

To detect the effects of PrP106–126 on cellular tubulin and microtubule, SK-N-SH cells were treated with 200 µM PrP106–126 or SCR peptide. Our result showed there is no significant changes in the expression levels of cellular tubulin after exposure to PrP106–126 for 6 or 12 hours(Fig. 6A and B). Immunofluorescence staining with an anti-tubulin antibody was performed on cells exposed to various agents were to visualize the alteration of cytoskeletal network. In the cells treated with DMSO and SCR peptide, large amounts of long fibril-like microtubule structures were observed in the cytoplasm. In cells exposed to PrP106–126, the fibril-like structures disappeared and only variably sized granular elements were observed (Fig. 6C). These results suggest that, under our experimental condition, cytotoxic peptide PrP106–126 induces a rapid disorganization of cellular microtubule network before significantly reducing tubulin levels.

### Increase of calpain in PrP106–126 treated cells

**Figure 7:**
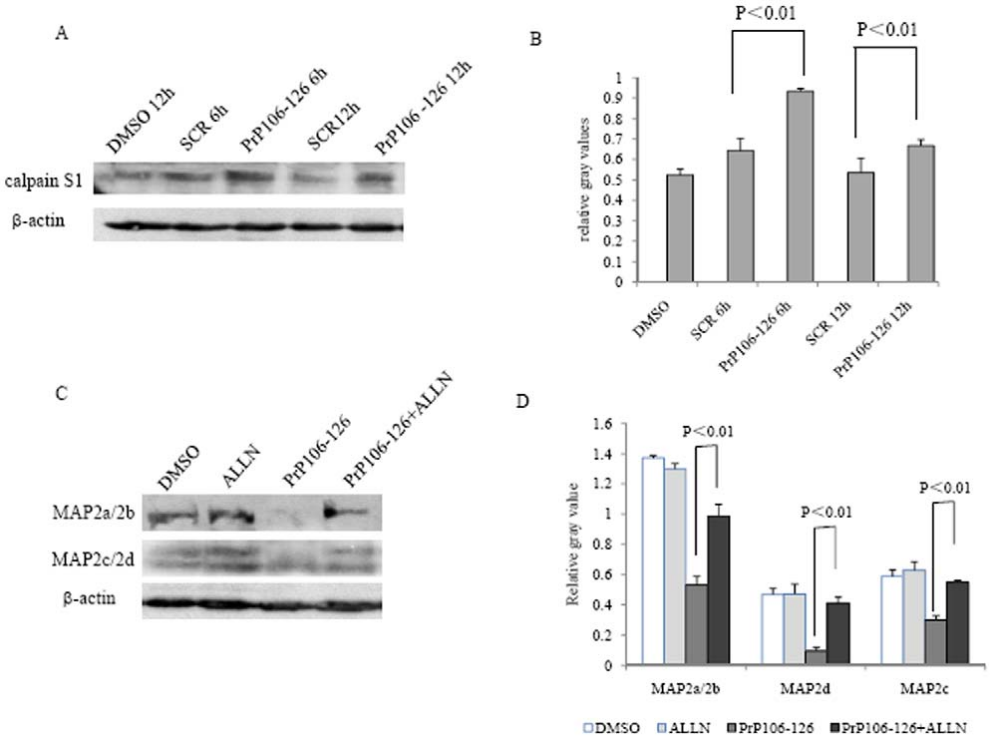
Analyses of calpain levels in PrP106–126 treated SK-N-SH cells. A. Western blots of calpain in cells exposed to DMSO, PrP106–126 or SCR. B. Quantitative analyses of the gray numerical values of calpain. The average relative gray value is calculated from three independent blots and presented as mean ± S.D. Statistical differences are illustrated as P<0.01. C. Western blots of MAP2 in the cells treated with PrP106–126 alone and PrP106–126 plus calpain inhibitor ALLN. D. Quantitative analyses of the gray numerical values of MAP2a/2b, MAP2d and MAP2c. The average relative gray value is calculated from three independent blots and presented as mean ± S.D. Statistical differences are illustrated as P<0.01.

Calpain is believed to cause degradation for a series of cell cytoskeletal proteins. To gain insight into the possible relationship between MAP2 and calpain, the level of calpain in the SK-N-SH cells treated with PrP106–126 was evaluated by Western blots. Clearly stronger calpain S1 signals were observed in the cells exposed to PrP106–126 (Fig. 7A), showing a significant increase (P<0.01) compared to cells treated with SCR peptide and DMSO (Fig. 7B). To detect the influence of inhibition by calpain on the MAP2 levels, calpain inhibitor ALLN was introduced into the cells exposed to PrP106–126. Compared with the remarkable reduction of MAP2a/2b in the cells exposed to PrP106–126, the cellular MAP2a/2b levels in cells exposed to PrP106–126 plus ALLN were almost as high as that of control of DMSO cells (Fig. 7C and D). ALLN treatment alone did not affect the level of MAP2 or calpain (data not shown). These data highlight that exposure of the cytotoxic peptide PrP106–126 to the cultured cells induces an increased calpain level, whcih might be responsible for the reduction of MAP2.

### Increase of calpain in the brains of 263K-infected hamsters

**Figure 8:**
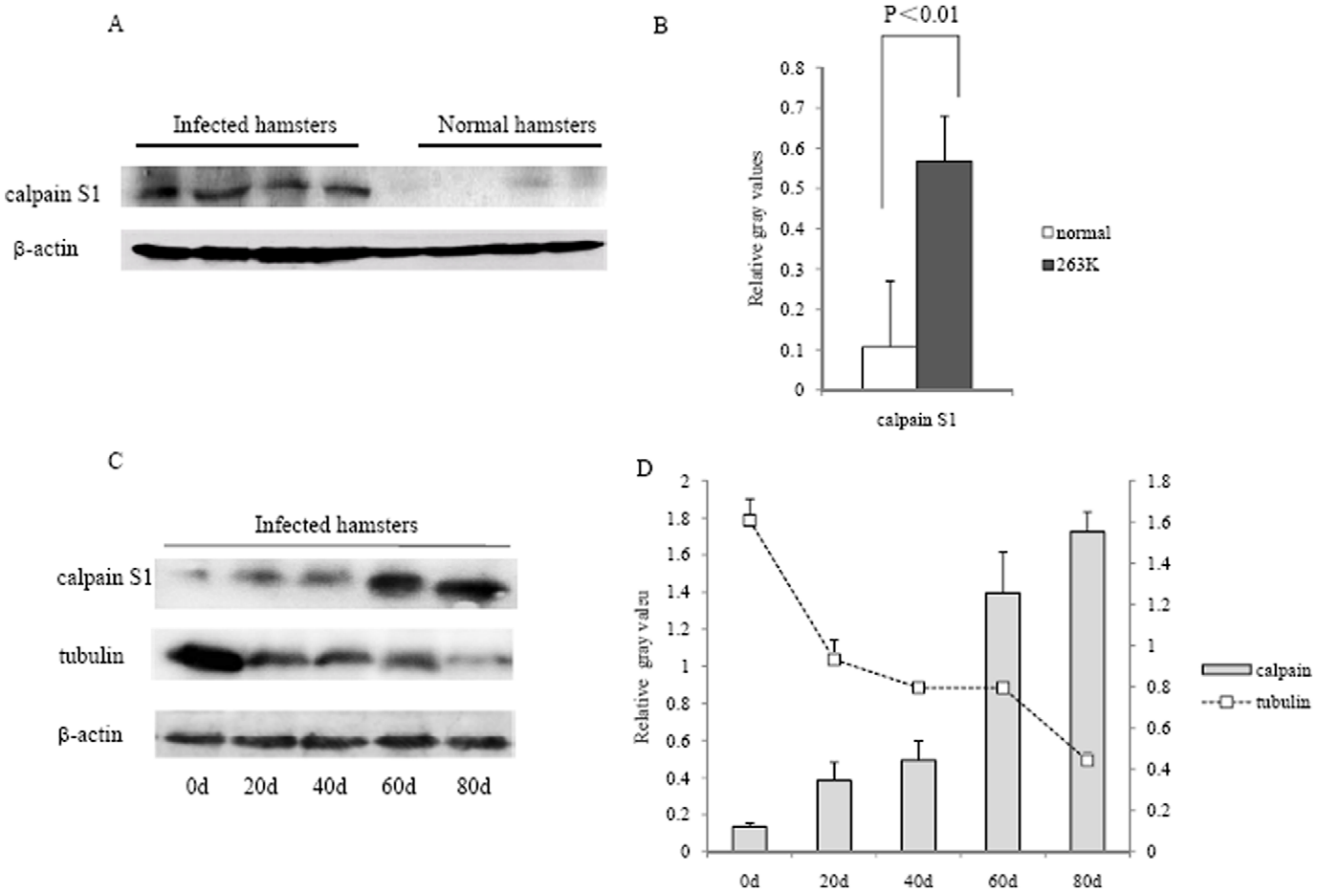
Analyses of calpain levels in the brain tissues of 263K-infected hamsters. A. Western blots of calpain in the infected and normal hamsters. B. Quantitative analyses of the gray numerical values of calpain. The average gray values were calculated from four infected hamsters or or four normal after normalized with that of individual β-actin and presented as mean ± SD. Statistical difference is illustrated as P<0.01. C. Western blots of calpain and tubulin in the brain tissues of 263K-infected hamsters on 0, 20, 40, 60 and 80 dpi. D. Quantitative analyses of the gray numerical values of calpain and tubulin vs that of individual β-actin. The average relative gray value is calculated from three independent blots and presented as mean ± S.D.

We also tested the potential alterations of calpain in the brain tissues of TSE experimental animals. The protein level of calpain was much higher in 263K-infected hamsters than normal controls (Fig. 8A) and quantification revealed a significant difference (P<0.01) between two groups (Fig. 8B). To determine the dynamic changes of calpain during prion infection, levels of calpain in the brains of 263K-infected hamsters collected during incubation period were comparatively analyzed by Western blot. An obvious time-dependent increase of calpain was observed along with the prolonging incubation, accompanying with a gradually decrease of tubulin signals (Fig. 8C and D). These data indicates a more active status of calpain in the brain tissues of TSE infected animals.

### Inhibition of calpain activity reversed the PrP106–126 induced destruction on microtubule structure and cytotoxicity

**Figure 9:**
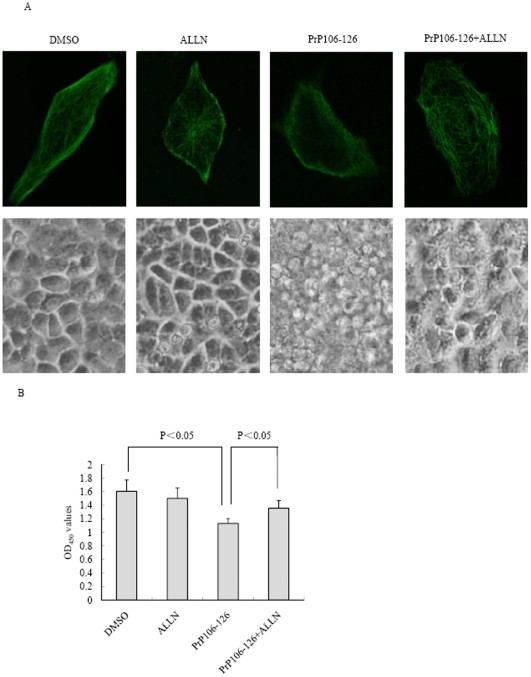
Inhibition of calpain activity reversed the PrP106–126-induced destruction on microtubule structures and cytotoxicity. A. SK-N-SH cells were treated with 200 µM of PrP 106–126 with or without calpain inhibitor ALLN for 12 h. Immunofluorescence images of cellular microtubule structure (upper) and morphological analyses of cells (lower) were shown. B. Cell viability after exposed to PrP106–126 with or without ALLN. The average data of each preparation was calculated based on three independent experiments and represented as mean ± S.D. Statistical differences are illustrated as P<0.05.

To evaluate potential role of calpain in mediating the PrP106–126-induced destruction of microtubule structures network and cytotoxicity, SK-N-SH cells were pretreated with 20 µM ALLN for 4 h and then cells were exposed to PrP106–126 for 12 h. As expected, cellular microtubule was significantly impaired when cells were treated with PrP106–126. Importantly, the destructive effects of PrP106–126 was substantially reversed in the cells pretreated with ALLN. ALLN alone did not change the cellular microtubule structure (Fig. 9A). Morphological analyses revealed more normal cells after the treatment of PrP106–126 plus ALLN than that of PrP106–126 alone (Fig. 9A). CCK8 tests identified that the cell viability of the reaction of PrP106–126 plus ALLN was partially, but significantly (P<0.05) improved compared with that of PrP106–126 (Fig. 9B). This result suggests that inhibition of calpain activity is able to partially reverse PrP106–126 induced cytotoxicity.

## Discussion

In this study we have demonstrated that levels of MAP2 in the CNS tissues of a scrapie experimental rodent model and in the postmortem brains of the patients of genetic prion diseases are significantly reduced, highlighting a common phenotype in TSEs. Mammalian brain MAP2 is composed of at least four isoforms, designated as MAP2a, 2b, 2c, and 2d, varying from the electrophoresis mobility on SDS-PAGE. Through alternative splicing of a pre-mRNA transcribed from a single gene located at chromosome 2 in human, multiple MAP2 isoforms are translated and presented in the CNS tissues [22]. In line with the observations that MAP2a/2b are the most abundant forms in adult brains [23], [24], clear MAP2a/2b bands are detected in the brain tissues of adult hamsters and human. Meanwhile, weak MAP2c/2d bands in the brain lysates of adult hamsters are consistent with that LMWMAP2 are mainly expressed during early developmental stages, which may decrease dramatically in the brains of mice postnatal 20 days [25].

Earlier studies have found that MAP2 polyclonal antibody might label abnormal neurites around senile plaques [26], and accumulation of soluble Aβ oligomers in a transgenic mouse model of AD results in a decrease in MAP2 labeling before these mice develop plaques [12], suggesting a possible role of MAP2 in neurodegenerative diseases. As a biomarker for neurons, reduction of MAP2 at the terminal stages of TSEs is reasonable. However, compared with the alteration of another neuronal marker NSE that reduces but usually remains detectable in prion diseases with conventional techniques (such as Western blot and IHC), the reduction of MAP2 isoforms are much significant and almost no specific signal could be detected by Western blots. In addition, our dynamic analysis of scrapie-infected rodents identify that the disappearance of MAP2 seems to occur at the early stage of infection, as it reduces to undetectable level in the 20 dpi samples. At this time, pathological PrP^Sc^ start to deposit in CNS tissues, which are visible in the assays of immunohistochemistry, but undetectable by Western blots [27]. Springer and his co-workers have proposed that MAP2 decreased dramatically within 1 hour after traumatic spinal cord injury, which is believed that as somatodendritic compartments of neurons, MAP2 may be especially vulnerable during diseases of nervous system [28]. Although we do not know the exact time when MAP2 starts to drop-down during prion infection, our data here indicate that the expression of MAP2 in neurons is more vulnerable during the progression of prion diseases, which may reflect that both the biological function of MAP2 in neurons and the cluster of MAP2 positive neurons are affected. Moreover, the reduction of MAP2 isoforms has been reproduced in a cultured neuroblastoma cell line exposed to the cytotoxic peptide PrP106–126 in this study. It emphasizes again the possibility of the disruption of MAP2 exposed to pathological prion.

Previous reports have suggested that PrP106–126 can bind to microtubules [29] and electron microscopy has confirmed that PrP106–126 can enter the cytosol and bind to microtubules when applied to cultured cerebellar cells [30]. PrP106–126 also shows the ability to inhibit tubulin polymerization by preventing the interaction of tau with tubulin in a cell-free system [31]. As a PrP interacting partner, the polymerization of tubulin and the cellular microtubule structures can be impaired directly via interacting with a couple of CJD-associated PrP mutants [16], [18], or indirectly via influencing on other microtubule associated proteins, such as tau [32] and TPPP [33]. Tubulin level has also been proved to be decreased in the brains of different TSEs [16]. These data strongly indicate that the disruption of microtubule structure is one of milestones in the pathogenesis of prion disease. As an important component of MAPs, it is reasonable to hypothesize that the reduction of MAP2 will be another pathway to demolish microtubule structures in TSEs. Additionally, our data here illustrate that the reduction of MAP2 and disruption of microtubule structure occur earlier and more obvious than the changes of tubulin and deposits of PrP^Sc^, highlighting that change of MAP2 is an early and persistent event in progression of TSEs. In fact, as the importance of cytoskeleton in the functions of neurons, interference with microtubule network is likely to lead to apoptosis with the impairment of axonal transport of various vesicles and organelles [34].

Calpain is a ubiquitous calcium-dependent protease which is essential for physiological neuronal function and the activation of calpain can be triggered by calcium influx and oxidative stress [35]. MAP2 isoforms are the substrates for the activated calpain [13] and the neuronal apoptotic events involved in alterations of the microtubule network are consequent to calpain activation [36]. Calpain-dependent degradation of MAP2 is known as an early response to traumatic [28], focal ischemic brain injuries [37] and glutamate excitotoxicity [38]. In addition, there are considerable evidences that an increased activity of calpain associated with impaired calcium homeostasis may be involved in AD development [13], [39], [40]. Recent study has suggested that the apoptosis induced by the peptide PrP106–126 is possibly through two biochemical pathways, caspase and calpain [41]. Moreover, our assays illustrate a time-dependent increase in calpain level during scrapie infection, along with a gradually decrease in tubulin level. Inhibition of calpain activity not only reverses the reduction of cellular MAP2 level, but also partially rescues PrP106–126 induced cytotoxicity. It is reasonable to speculate that the abrupt reduction of MAP2 in brain tissues is associated with the rapid increase of calpain in prion diseases. Actually, disturbance of the calpain system in CNS tissues has been observed in some neurodegenerative diseases including Alzheimer's [42], Parkinson's [43] and prion diseases [41]. The central role of microtubules and microtubule transport even make them an appealing target in developing therapy of neurodegenerative diseases. Neuronal death due to PrP106–126 is reduced following pharmacologic calpain inhibition [39] and MAP2 degradation is reversed by pretreated with calpain inhibitor in neuronal cells exposed to Aβ [13]. Hence, further characterizations of the role of calpain and MAP2 in the pathogenesis of prion diseases are specially needed.
